# Effects of Polymeric Media-Coated Gynosaponin on Microbial Abundance, Rumen Fermentation Properties and Methanogenesis in Xinjiang Goats

**DOI:** 10.3390/ani12162035

**Published:** 2022-08-10

**Authors:** Peng Li, Irum Mohd Mehmood, Wei Chen

**Affiliations:** 1 School of Agriculture, Ningxia University, Yinchuan 750021, China; 2 Faculty of Agriculture, Cairo University, Cairo 12613, Egypt

**Keywords:** gynosaponin, methanogenesis, fermentation properties, microbial abundance, goats

## Abstract

**Simple Summary:**

Saponins are famous for their anti-methane effects; however, due to their structural differences that have different fates of antimicrobial activity in the rumen, their effects are not consistent in the literature. Thus, the current study used polymeric media-coated gynosaponin (PMCG) to estimate the time-dependent effects on the methanogenesis, rumen fermentation properties and microbial abundance in goats. We used Xinjiang goats (n = 8) that were divided into two groups, i.e., the PMCG group (8 g/kg DMI) and a control group (0 g/kg DMI). Ruminal contents were analyzed for rumen fermentation properties and microbial abundance. Protozoa numbers were counted to calculate the abundance of methanogens, rumen fungi and cellulolytic bacteria using real-time PCR. The results indicated that PMCG significantly reduced the methane production, and decreased the acetate to propionate ratio and total VFA concentration. The protozoa numbers and gene copies of methanogens, total bacteria and F. succinogens relative to the 16 s rDNA were all slightly decreased. Overall, the addition of PMCG had an inhibitory effect on methane production.

**Abstract:**

Gynosaponin is known to modulate rumen methanogenesis and microbial fermentation characteristics in ruminants. The current experiment aimed to determine the time-dependent effects of intraruminal polymeric media-coated gynosaponin (PMCG) supplementation on the methanogenesis, rumen fermentation properties and microbial abundance in Xinjiang goats. Eight goats were used in a 2 × 2 crossover arrangement with a PMCG group (8 g/kg DMI) and a control group (0 g/kg DMI). The experiment was divided into four phases, each lasted 21 d. Ruminal contents were obtained for analysis of rumen fermentation properties and microbial abundance. Protozoa numbers were counted by microscope and the abundance of methanogens, rumen fungi and cellulolytic bacteria were quantified by real-time PCR. The results indicated that PMCG significantly reduced methane production (*p* < 0.05) during the first two phases but this increased to baseline again during the last two phases. Meanwhile, the concentration of acetate decreased remarkably, which resulted in a significant reduction in the acetate to propionate ratio and total VFA concentration (*p* < 0.05). However, other rumen properties and dry matter intake were not affected (*p* > 0.05). During the first and second phases, the protozoa numbers and gene copies of methanogens, total bacteria and *F. succinogens* relative to the 16 s rDNA were all slightly decreased, but the statistical results were not significant. However, the ruminal supplementation of PMCG had little effect on other tested microbes. Accordingly, it was concluded that the addition of PMCG had an inhibitory effect on methane production probably due to a decline in methanogen numbers.

## 1. Introduction

Global warming is one of the most serious international environmental problems. In recent decades, global warming brought about a series of disasters to the global natural world and ecological environment. It is documented that the key motive for global warming is the growing concentration of greenhouse gases (GHGs), including methane (CH_4_), nitrous oxide (N_2_O), and carbon dioxide (CO_2_), etc. Methane is strongly believed to contribute to global warming and climate change because methane has 23 to 25 times more global warming potential than carbon dioxide [1] Apart from the industrial processes, the livestock sector emerges as the largest source of methane emissions from the agricultural sector because methane from farm animals accounted for 38% of the greenhouse gases produced [2], in which ruminants contribute to approximately 90% of the greenhouse gas emissions [3]. Moreover, in the ruminal fermentation process, 2–15% of ingested gross energy is converted to methane [4]. Therefore, making every effort to inhibit the methane production from ruminants not only can improve feed efficiency, but also mitigate the greenhouse effects and bring outstanding environmental advantages [4]. Gut methane is produced by methanogenic archaea as a byproduct of feed fermentation and is eructated as waste from the animal into the atmosphere [5]. Manipulation of the rumen microbial ecosystem for improving feed digestibility and decreasing methane emissions by livestock are some of the most significant aims for animal nutritionists [6].

Dietary modulations to relieve methane emissions include: decreasing the fermentation of organic matters in the rumen, diverting hydrogen away from methane emission during ruminal fermentation and shifting the site of digestion from the rumen to the intestines, etc. [7,8]. Regulation of rumen fermentation to enhance feed efficiency is often achieved by feeding sub-therapeutic levels of antibiotics to ruminants [9]. However, there are increasing concerns over the use of antimicrobials in the farm animal industry due to its great risk to human health [10].

In the last decade, secondary plant metabolites, which include saponins, have emerged as an alternative to substitute antimicrobial feed additives [11,12,13]. Saponins are the naturally occurring glycosides found in various forms and structures in all legume plant species. These have sugar moiety in the structure which results in their classification as triterpenoids and steroids [14]. Saponins, being a part of the plant defense system, were reported to overturn methane production, decrease rumen protozoa counts, and modify fermentation patterns [9]. They were also reported to have many pharmacological activities including effects on gastrointestinal absorption, metabolism, ruminal digestion and enzyme activity (as reviewed by [15]). Saponin-rich *Brachiaria decumbens* significantly lowered gas production, acetic acid, and total VFA concentrations in sheep as compared to the control group [16]. However, the inconsistent effects of saponins were reported in the literature due to their structural differences that produce different fates of antimicrobial activity in the rumen (as reviewed by [17]). Therefore, in recent years, researchers have been trying to use smart antibacterial coatings to improve the efficacy of antimicrobials and microbial modulators [18]. The present study is the first attempt in using polymeric media-coated gynosaponin (PMCG) to investigate the time-dependent effects on methane emission, fermentation properties and microbial abundance in the rumen of Xinjian goats. The antimicrobial activity of polymeric material is well acknowledged in the literature [19]. In a study, polymeric calcium pectinate-coated urea modulated the protozoa population and caused the slow release of urea [20]. In the light of the aforementioned studies, we hypothesized that PMCG might have potential effects on methane emission.

## 2. Materials and Methods

### 2.1. Goat Trials

Eight male Xinjian goats (average body weight of 21 ± 2 kg, age 6.0 *±* 1.0 months) were randomly allocated into two groups, four goats for each group. The goats were housed in individual pens in a naturally ventilated house. Feed (milled, 0.9 mm) was given in equal portions twice a day at 08:00 and 17:00; while water was available at all times. The ingredients and nutritional composition of the basal diet are presented in Table 1. The amount of basal diet offered was calculated and adjusted daily to leave about 10% of leftovers or a thin layer in the feed bunk. Refused feed was taken together daily, collected at a dry place, and used to weigh every three days to calculate feed intake. The experiment was approved by the Institutional Animal Ethics Committee and was carried out according to a 2 × 2 crossover arrangement (two periods) with a PMCG group (8 g/kg DMI) and a control group (0 g/kg DMI). The saponin dose was chosen based on the previously published literature [21,22]. PMCG was kindly provided by Prof. Tong Zhang, Ningxia University, China, prepared in his lab. It was prepared using the spin coating method, which is known as the best method to obtain high-quality polymeric coatings [23]. PMCG was dissolved in 60 mL physiological saline and intraruminally given by an injector just before each feeding time. The control group was also given 60 mL physiological saline intraruminally to keep the same condition. The integral time of the experiment was about 84 days for each period, which was divided into four phases with 21 days in each phase. Before the beginning of each period, there were 21 days of basal diet adaptation time and 7 days of methane measuring and sampling time. After each feeding trial for 14 days, the goats were transported to respiratory chambers to measure methane production. Each measurement lasted for 60 h. The first 12 h within the chambers was considered as adaptation time; measurements were recorded for 48 h. After the methane measurement, rumen contents were obtained at 0 (immediately prior to feeding), 2, 4 and 8 h after feeding in the morning for analysis of ruminal fermentation properties and 0 h samples were also used for analysis of rumen microbial abundance. The rumen fluid was collected via the fistula in a bottle, previously kept warm and filled with CO_2_, and then filtered through two layers of cheesecloth immediately after collection. It was stored at 39 °C until used as inocula [24]. Protozoa numbers were counted by microscope and gene copies of rumen bacteria, methanogens, fungi, *Ruminococcus flavefaciens*, and *Fibrobacter succinogenes* were tested by RT-PCR, as a proportion of the total rumen bacterial 16 s rDNA (gene encoding ribosomal RNA) [25,26].

### 2.2. Analytical Procedures

#### 2.2.1. Feed Intake and Methane Measurement

Offered and refused feeds were recorded once every 3-day period for measuring the feed intake. During the two successive days when goats were housed in the simple open-circuit respiratory chambers, which were cuboids (1.0 × 1.2 × 1.2 m) constructed with steel bar around PVC (Polyvinyl chloride) glasses and cement concrete surface, methane concentration from the inlet and the outlet of each chamber were measured. The LumaSense Photoacoustic Gas-Monitor INNOVA 1412i and Multi-Sampling 1309 were used to measure the methane concentration, which are highly accurate, reliable and stable quantitative gas monitors, based on the photoacoustic infrared detection method measurement principles. The volume of the air which flowed through the chamber was documented by the Lutron AM-4202 Anemometer (from Taiwan) once an hour. The quantity of methane was measured considering the methane concentration from the inlet and outlet data, and the air that passes through the compartment. Prior to the measurement, methane volume from the chambers was recognized to calibrate the compartment by using the typical methane gas intake (99.99%, 0.83 cm^3^/s; [26]). The calculated methane output of these four chambers were 0.97, 0.93, 0.94 and 0.96, respectively. The methane production was calculated by the following equation: VCH4 = (P − P0) × V/R, where P and P0 are the methane concentration (*v*/*v*) of the air samples taken from the outlet and inlet, respectively; V is the volume of air that run through the chamber (L); and R is the recovery of methane in the chamber.

#### 2.2.2. Sample Collection and Chemical Analyses

After the methane measurement, rumen contents were collected on two consecutive days at 0 (immediately prior to feeding), 2, 4 and 8 h after the morning feed. The samples were collected from various sites of the rumen, composited and squeezed immediately through two layers of cheesecloth [22]. The pH of rumen samples was determined immediately using a pH meter (Laqua F-72, Horiba Scientific Co., Tokyo, Japan). The samples were then stored at −20 °C for further analyses of microbial protein ammonia N, and total volatile fatty acid (TVFA), using methods described by [27].

#### 2.2.3. Protozoa Counting, total DNA Extraction and Real-Time Quantitative PCR

The samples collected at 0 h (immediately prior to feeding) were used for analysis of rumen microbial abundance. Protozoa numbers were counted by microscope. Total DNA was extracted from rumen contents by cetyltrimethyl ammonium bromide (CTAB) and Phenol–Chloroform–Isoamyl alcohol method [26]. The samples were centrifuged at 13,000× *g* for 10 min and the supernatants were thrown away. The sediments were resuspended in 1 mLCTAB buffer (0.1 M Tris HCl, pH 8.0, 1.4 M NaCl, 0.02 M EDTA, 2% CTAB), beaten for 2 × 120 s at a speed of 6.0 using bead-beater equipment (FastPrep-24, India) and incubated for 20 min at 70 °C. Phenol–Chloroform–Isoamyl alcohol was added and the samples were vortexed for 1 min and centrifuged for 10 min (at 13,000× *g*) at ambient temperature. The upper aqueous layers were carefully removed; this step was repeated several times until the liquid was totally clear. After centrifuging for 10 min at 13,000× *g*, the resultant DNA pellets were washed with iced 70% ethanol (1 mL, −20 °C), again centrifuged for 20 min at 13,000× *g* with ethanol removed. Then, it was re-suspended in 50 mL TE buffer (pH 8.0) and the DNA was dissolved at 70 °C, for 5 min using thermomixer compact then stored at −20 °C. Species-specific primers of methanogens (*mcrA*), total bacteria, fungi, *R. flavefaciens* and *F. succinogenes* were used to amplify the partial 16 s rDNA regions listed in Table 2, as described previously [28,29].

The real-time quantitative PCR was performed using the ABI 7300 real-time PCR system (Applied Biosystems, Waltham, MA, USA). Amplification condition was as follows: one cycle at 95 °C for 10 s for initial denaturation and followed by 40 cycles of 95 °C for 15 s and 60 °C for 1 min. The amplification conditions and reaction mixture were adopted from the study of [26]. Measurements were carried out in triplicate for each run including a negative control and the mean values were calculated. Populations of total bacteria, fungi, methanogens, *F. succinogenes* and *R. flavefaciens* were expressed as a proportion of total rumen bacterial 16 s rDNA. DNA concentration of the PCR product measured on GeneQuant pro RNA/DNA calculator directly related to copy numbers using the following equation:

Copy number/µL = (C/X) × 0.912 × 10^12^ (1)

C: DNA concentration measured (ng/µL); X: PCR fragment length (bp/copy).

### 2.3. Statistical Analysis

Initially, all data were calculated by Microsoft excel software and statistical analysis was carried out using General Linear Model (univariate) and analysis of variance (ANOVA) in the statistical software package SPSS (version16.0; SPSS Inst. Inc., Cary, NC, USA). A factorial arrangement, in which the first factor was the treatment and the second factor was the phase, was employed. The statistical model used was: Yijk = µ+ Di + Pj + Tk + (PT)ik + eijk, where Yijk = observed values, µ = grand mean, Di = effect of the day of sampling, Pj = effect of phase, Tk = effect of experimental treatment, (PT)ik = interaction effect between phase and treatment, and eijk = residual error. When a parameter was found significant at *p*  < 0 .05, it was followed by a post-hoc test.

## 3. Results and Discussion

### 3.1. Effects on Feed Intake and Methane Emission

The intraruminal supplementation of PMCG had little effect on feed intake at different phases (Table 3). This may be because the diet in our experiment was formulated to meet the maintenance requirement of goats. This may be further explained by considering the factors that affect saponin solubility and functionality including pH, temperature and composition of gut contents, chewing or rumination, feed degradation, feeding time, and the rate of diet passage through the gut [30,31]. The available literature exhibited that the dietary inclusion of saponins in farm animals had no deleterious effects on feed intake [21,32,33,34,35]. The result of the 15-day in vivo experiment, which was carried out by [36], showed that the addition of a sarsaponin 0.12 g/kg diet decreased the methane emission by 7.1%, however, sarsaponin had no effect on feed intake [37]. Wang et al. [9] reported that feed intake did not change with decreasing methane production by saponins.

**Figure 1:**
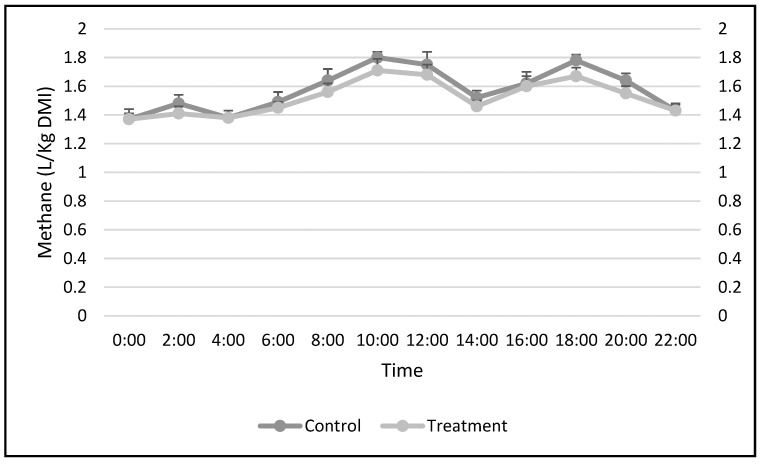
Changing trend of the methane production of goats in a day. The methane was measured using the LumaSense Photoacoustic Gas-Monitor INNOVA 1412i and Multi-Sampling 1309 gas monitor systems. The measurement was carried out for 48 h, however same trend was observed for both days. Error bars represent the SEM (standard error mean).

During the 48 h methane measurement, the same changing trend was observed in daily methane production in the treatment and the control group which was high around 1 to 4 h after the animals were fed and then lowered slowly until the next feeding (Figure 1). This trend was similar to the experiment conducted by [21,38]. Compared with the control group, methane emission of the treatment group decreased by 6.08% and 6.76% (*p* < 0.05) during phase 1 and phase 2, but increased to baseline again during the last two phases (Table 3).

Lots of research has found inhibition effects of saponins on methane production. In the 21-day and 60-day in vivo experiments, which were conducted by [21,38], tea saponins supplemented with sheep and lamb diets reduced methane production by 8.71% and 27.2%, respectively. Guo et al. [39] reported that tea saponin significantly reduced methane production by 8% after 24 h of incubation compared with the untreated control group. Xu et al. [40] reported that methane proportion and production were both significantly decreased by *Yucca schidigera* extract addition. Wang et al. [41] demonstrated that in 48 h incubation of 0.5 mg/mL *Yucca schidigera* saponin, methane production was lowered by 15% in the treatment group compared with the control. [36] found that sarsaponin inhibited methane emission by 6.7% when added to a goat’s diet for 14 days. Pen et al. [42] also observed the methane inhibition effects of sarsaponin during another in vivo experiment. Alfalfa saponins were also known to reduce methane production linearly at 24 and 48 h incubation [43]. However, there was some research that showed different results. Holtshausen et al. [44] reported that the addition of *Quillaja saponaria* 10 g/kg diet (forage (F): concentrate (C) = 51:49) has no effect on methane emission during a 28-day cattle trial. Tea seed (*Camellia sinensis* L.) saponins did not show any inhibitory effect on methane production in cattle [22]. In the presence of *Enterolobium cyclocarpum* fruits (saponin, 19 g/kg), methane emission was increased by 14% in a 10-day Rusitec experiment [45]. Among the above studies, the different types of saponins showed different effects in different experiments, even the same saponin tested in different experiments indicated various results. All the inconsistencies among the studies about the effectiveness of adding saponins to the diet on methane production may have resulted from many factors, including the type and dose of saponins, the type and composition of the diet, etc. [30,31]. Although many experiments were conducted to investigate the effects of different saponins on rumen methane production, they were all in vitro or short-term in vivo experiments. In our current study, we observed that methane emission was suppressed by the intraruminal addition of polymeric media-coated gynosaponin, but this effect can not last long. This could be correlated with the study of [25], who reported that methane-producing organisms (methanogens) were suppressed up to the 6th day of treatment and then increased for the rest of the experimental period (day 30). Diet is the most important factor that modulates the rumen microbiota, and any change in the diet may significantly shift the microbial communities. It could be hypothesized that in the current experiment, the PMCG treatment made an instant shift in microbial communities that results in the suppression of methane emission. Moreover, it was reported that a quantitative increase in one type/group of microorganisms in the gut may modify other gut microbiota in two ways: antagonism and competitive exclusion. Dominant microorganisms in the gut produce many compounds (bacteriocins) such as lactoferrin, organic aids, and hydrogen peroxide that inhibit the growth of many other microorganisms [25,46]. For instance, Chen and Weimer [47] reported that rumen bacteria *R. flavefaciens* and *F. succinogenes* were suppressed by the inhibitors produced by *R. albus*.

### 3.2. Effects on Fermentation Properties

Microbial protein, amino-nitrogen, pH value and volatile fatty acids are important parameters that play important roles to balance the rumen inner environment. Concentrations of microbial protein, amino-nitrogen and pH value of the rumen were little affected by the addition of PMCG during the integral experiment. MCP is known to be important for growth and production in ruminants. The little effect on MCP recorded in the current study suggested the improved activity of saponin-stimulated and -utilizing organisms by the PMCG treatment, as suggested earlier [48]. The concentration of acetate in the treatment group was significantly (*p* < 0.05) lower than the control group during phase 1 and phase 2. However, in phases 3 and 4, the acetate concentration increased rapidly and recovered to the same level as the control group. The propionate concentration of the treatment group was somehow higher than the control group in all phases but there were significant differences among them. The above changes resulted in a significant (*p* < 0.05) reduction of the acetate/propionate ratio in the first two phases. The TVFA concentration also decreased significantly (*p* < 0.05; Table 4).

Wina et al. [49] demonstrated that molar proportions of acetate and butyrate decreased by 5.6 units and 2.9 units, respectively, whereas propionate improved significantly from 20.8% (control) to 30.1% (treatment) in an in vitro fermentation by 4 mg/mL methanol extract of *Sapindus rarak*. Patra et al. [50] reported that concentrations of acetate and total volatile fatty acids did not show any relationship (*p* > 0.1) with changes in methane due to saponins. However, propionate production raised linearly with a growing inhibition of methane, which resulted in a linear reduction in acetate:propionate (A/P) with declining methane production. [33] reported that with supplementation of *Y. schidigera* extract 16.4 g/day (F: C = 3:2) in a sheep’s diet, methane production decreased without affecting the acetate to propionate ratio. Tea saponin increased (*p* < 0.05) the molar proportion of propionate from 21.5% to 24.1% and reduced (*p* < 0.05) the ratio of acetate to propionate from 3.0% to 2.6% [39]. The result of a 15-day sheep experiment conducted by [9] showed that the addition of a saponin 0.13 g/kg diet (F: C = 3:1) lowered the methane production and acetate/propionate ratio but increased the TVFA. [35] reported that saponins of *Gliricidia sepium* and *Enterolobium cyclocarpum* exerted a linear effect (*p* < 0.05) on the butyric acid and acetic:propionic acid ratio without affecting methane production. Supapong et al. [51] demonstrated that the propionate proportion of TVFA was increased by *Delonix regia* seed saponins. Due to the different structures of saponins, the saponins displayed different bioactive properties [42,52]. The addition of saponins might increase propionate production as a result of the rechannelling of hydrogen from methane to propionate and declining acetate: propionate, which is valuable nutritionally for ruminants. The formation of acetate in the rumen results in great quantities of hydrogen and depends on the availability of falling equivalents such as NAD+ [53]. The high NADH/NAD+ ratio and high fractional pressure of hydrogen in the rumen due to the inhibition of methanogenesis may result in the decline in acetate production [53,54]. The reduction of acetate concentration and acetate to propionate ratio in the current study may be due to the above reasons. Our study suggested evaluating the physiological effects of PMCG treatment including the effect on milk production and composition in dairy animals because VFA is the precursor of milk fat synthesis and various ratios of VFA have a significant contribution to glucose synthesis, milk composition, milk fats, etc [10,55].

### 3.3. Effects on Rumen Microbial Population

The microbes tested in this experiment responded differently to the intraruminal supplementation of PMCG (Table 5). The relative abundances of Ruminococcus and Fungi were little affected potentially because of the high forage diet in our experiment, however, the abundance of total bacteria and Fibrobacter were slightly decreased (*p* > 0.05). The mcrA gene copies of methanogens in the treatment group were respectively lower than the control group by 22.4% and 28.0% during the first and second phases, and compared with the control group, the numbers of protozoa also decreased by 14.7% and 12.3% separately in the treatment group during these phases but rebounded to baseline level again during the last two phases. However, these differences may be due to individual variations, as all these results did not show any statistical significance.

Bacteria are the most frequent microorganisms in the rumen and play a vital role in dietary fiber degradation [56]. Earlier work has shown that fiber-degrading bacteria were more susceptible to saponins as compared to starch-degrading bacteria [26,41]. This may moderately explain the present result that the fiber-degrading bacterial organisms were obviously affected as compared to the entire bacterial population that was less affected.

Methanogens are responsible for methane production in the rumen. Methanobrevibacter phylotypes are the dominant methanogens in ruminant livestock [57]. Several studies have reported an inhibitory effect of saponins on methanogens in the rumen. The methane-suppressing effects of saponins are apparently a direct action against the rumen microbes involved in methane emission including protozoa and methanogens [58]. In co-culture systems of methanogen with fungi, gynosaponins significantly reduced methane concentration and inhibited methanogen growth [59]. In the in vitro fermentation experiment conducted by Goel et al. [60], methanogen populations were reduced due to the addition of Sesbania saponins by 78%, fenugreek saponins by 22% and Knautia saponins by 21% with the rumen liquor collected from cattle. However, the addition of tea saponins at 0.4 mg/mL in the fermentation media with the sheep rumen fluid did not show inhibitory effects on methanogens [39]. Jadhav et al. [61] observed a maximum of 36% decline in in vitro methane released by the tea seed saponins, along with a 60.6% decline in the protozoal count. In another in vitro gas production system, the presence of 0.14 and 0.29 g/L of the *Trigonella foenum-graecum* seeds extract (34.5% saponins) did not reduce methanogen numbers. A decrease in the activities of methane-producing genes or rate of methane production per methanogenic cell by saponins was also proposed. It was a pity that we did not test the activity of microbes in the current study. Declines in methane emissions were ascribed to a drop in methanogen numbers and also may be because of a potential decline in the metabolic rate of methanogens. Wina et al. [49] observed that the toxicity of feeding saponins at high levels occurred in bacteria, fungi and protozoa. The study proposed that saponins bind with the sterols present on the surface of protozoans (which are absent in bacterial membranes) thereby causing a toxic effect on protozoa. Muetzel et al. [62] reported that saponins from *Sesbania pachycarpa* enhanced the growth of Ruminococcus, whereas the study of [41] stated the negative effects of saponins from Yucca on the Ruminococcus population. Both the Fibrobacter and Ruminococcus were not affected by tea saponins [21]. Some researchers found a microbial adaptation to the saponins. For example, Wina et al. [49] reported that *Ruminococcus flavefaciens, Ruminococcus albus,* and *Chytridiomycetes* (fungi) adapted to saponin when fed for 28 days to goats. It had been stated by many researchers that the methane inhibition by saponins was related to the reduction in protozoa numbers. Patra et al. [50] reported that each 1% suppression of protozoal numbers accounted for about 0.17% inhibition of CH4 by saponins. It was proposed by [5] that saponins are toxic to rumen protozoa thereby reducing methane production. Rumen protozoa were thought to be of significance in methane production because of the relationship of symbiotic methanogens with these protozoa [58]. Thus methane inhibition by saponins conceivably results predominantly from diminished protozoal populations. Methanogens associated with protozoa account for decreased methane production of about 9–25% [63] or as much as 37% [55]. The sterol-binding capability of saponins was implicated in the destruction of protozoal cell membranes [64]. Tea saponins reduced the protozoal numbers but had no effect on methanogens [27,39]. Reductions in the rumen protozoa population, which were reported with *Yucca schidigera* extract feeding [65], were complemented by increases in propionate and falls in rumen butyrate, which suggests methane production was reduced. In long- and short-term defaunated ruminants, methane was decreased by about 20% in the absence of protozoa [66]. In vitro, 24 h fermentation with fenugreek seeds 174 g/kg, decreased methane by 9.7% and protozoal numbers decreased by 56% [67]. Ref [34] found that *Sapindus saponaria* fruits supplemented with a sheep’s diet reduced the protozoal counts but increased the methanogen numbers. However, in a 10-day Rusitec experiment, an *Enterolobium cyclocarpum* fruit 200 g/kg diet increased methane production by 14% and protozoal numbers by 54% [45], whereas [33] described that inclusion of plant saponins reduced the methane emission in ovine without effecting the protozoa population, showing that the elimination of methanogens that are associated with protozoa is not the only source of methane elimination. Reduction in methanogen population did not always follow a pattern similar to protozoal numbers. An earlier study [68] suggested that steroidal saponins reduced total protozoal in continuous culture but, typically no changes in protozoa numbers were observed [10,44]. The differences among these results may depend on the different types and doses of saponins or the level and composition of diets.

## 4. Conclusions

From the current study results, it could be concluded that polymeric media-coated gynosaponin (8 g/kg) could mitigate methane production. Depression of methane production by PMCG may primarily inhibit methanogens and bacteria, resulting in decreased concentrations of acetate and the acetate to propionate ratio, which may result from the accumulation of hydrogen. The addition of PMCG reduced gene copies of ruminal methanogen and rumen protozoa numbers to some extent but had little effect on the other microbes. Mitigation of methane emissions could be attributed to the changing pattern of rumen microbes and fermentation characteristics.

## Figures and Tables

**Table 1:** Ingredients and nutritional level of basal diet offered to goats.

Ingredient	Content
Alfalfa	555
Corn	271
Wheat bran	89
Soybean meal	71
Ca_2_HPO_4_	6
Mineral Premix *	8
**Nutritional level**	
Dry matter	962
Organic matter	842
Neutral detergent fiber	434
Acid detergent fiber	259
Crude protein	172
Calcium	5.8
Phosphorus	3.3

* Premix contained per kg: 20.0 g Mg, 0.48 g Fe, 1.2 g Mn, 2.2 g Zn, 45 mg Se, 44 mg I, 50 mg, Co, 85,000 IU vitamin A, 16,000 IU vitamin D, 1800 IU vitamin E.

**Table 2:** PCR primers for real-time PCR assay.

Target Species	Forward/Reverse	Primer Sequence	Amplicon
Methanogens ^b^	F	TTCGGTGGATCDCARAGRGC	140
	R	GBARGTCGWAWCCGTAGAATCC	
Total bacteria ^a^	F	CGGCAACGAGCGCAACCC	130
	R	CCATTGTAGCACGTGTGTAGCC	
Total fungi ^a^ *R. flavefaciens* ^b^ *F. succinogenes* ^b^	F R F R F R	GAGGAAGTAAAAGTCGTAACAAGGTTTC CAAATTCACAAAGGGTAGGATGATT CGAACGGAGATAATTTGAGTTTACTTAGG CGGTCTCTGTATGTTATGAGGTATTACC GTTCGGAATTACTGGGCGTAAA CGCCTGCCCCTGAACTATC	120 132 121

^a^ [29]; ^b^ [28].

**Table 3:** Effects of PMCG on dry matter intake and methane emission of goats.

Item	Phase					Treatment			*p*-Value		
	1	2	3	4	SEM	Control	PMCG	SEM	P	T	P × T
**Dry matter intake (g/day)**	678	680.5	683.5	687.5	6.7	683	681.7	7.01	0.05	0.55	0.96
**Methane (g/kg DMI)**	23.95	23.95	24.85	24.80	0.28	24.8	23.9	0.37	0.02	0.01	0.41

SEM: Standard error mean. P: Phase, T: treatment; P × T: Interaction of phase and treatment.

**Table 4:** Effects of PMCG on rumen fermentation properties of goats.

Item *	Phase					Treatment			*p*-Value		
	1	2	3	4	SEM	Control	PMCG	SEM	P	T	P × T
**pH value**	6.29	6.27	6.33	6.36	0.03	6.32	6.31	0.01	<0.01	0.77	0.63
**MCP (mg/mL)**	1.03	1.01	1.02	0.89	0.02	0.98	0.99	0.03	<0.01	0.40	0.03
**NH_3_-N (mM)**	7.93	7.96	7.84	7.76	0.27	8.01	7.74	0.16	0.27	0.05	0.42
**VFA proportion (mM)**											
**Acetate**	72.01	72.00	71.67	72.86	0.56	72.57	71.75	0.78	<0.01	0.01	0.25
**Propionate**	17.34	17.48	17.38	16.89	0.25	16.93	17.83	0.22	0.01	0.15	0.85
**Butyrate**	7.10	7.02	7.59	7.66	0.16	7.28	7.44	0.10	<0.01	0.19	0.45
**Valeric acid**	0.41	0.44	0.47	0.44	0.01	0.44	0.45	0.01	<0.01	0.17	0.70
**Isobutyrate**	2.01	2.26	1.57	1.14	0.04	1.81	1.65	0.09	<0.01	0.04	0.88
**Isovaleric**	1.10	0.77	1.19	0.99	0.05	0.99	1.03	0.04	<0.01	0.47	0.63
**TVFA (mM concentration)**	62.51	63.08	67.30	67.60	0.07	66.03	64.22	0.96	<0.01	0.01	0.47
**A/P ratio**	4.12	4.16	4.18	4.36	0.05	4.33	4.08	0.07	0.01	<0.01	0.11

* MCP: Microbial crude protein concentration; NH_3_-N: Ammoniacal nitrogen; TVFA: Total volatile fatty acid; A/P: Acetate to propionate ratio. SEM: Standard error mean; P: Phase, T: treatment; P × T: Interaction of phase and treatment.

**Table 5:** Effects of PMCG on the rumen microbial population.

Item	Phase					Treatment			*p*-Value		
	1	2	3	4	SEM	Control	PMCG	SEM	P	T	P × T
**Methanogens × 10^8^ (copies/mL)**	1.82	1.84	2.34	2.22	0.46	2.25	1.85	0.39	0.61	0.21	0.95
**Protozoa × 10^5^ (counts/mL)**	3.96	4.01	4.77	4.47	0.19	4.49	4.11	0.28	0.01	0.08	0.68
**Fungi × 10^6^ (copies/mL)**	2.63	2.19	1.27	1.71	0.60	1.77	2.13	0.43	0.07	0.27	1.00
**Bacteria × 10^10^ (copies/mL)**	6.52	6.62	7.02	7.50	0.79	7.29	6.54	1.14	0.78	0.46	0.99
***R. Flavefaciens* × 10^8^ (copies/mL)**	2.90	2.94	2.78	2.41	0.49	2.71	2.81	0.56	0.93	0.88	1.00
***S. Succinogens* × 10^8^ (copies/mL)**	2.22	2.08	2.49	3.00	0.50	2.57	2.32	0.43	0.28	0.37	1.00

SEM: Standard error mean. P: Phase, T: treatment; P × T: Interaction of phase and treatment.

## Data Availability

Data may be provided by the authors upon request.
